# Characterization of Different Inflammatory Skin Conditions in a Mouse Model of DNCB-Induced Atopic Dermatitis

**DOI:** 10.1007/s10753-023-01943-x

**Published:** 2023-12-27

**Authors:** Rebecca Riedl, Annika Kühn, Yvonne Hupfer, Betty Hebecker, Lukas K. Peltner, Paul M. Jordan, Oliver Werz, Stefan Lorkowski, Cornelia Wiegand, Maria Wallert

**Affiliations:** 1 Department of Dermatology, Dermatological Research Laboratory, Jena University Hospital, 07747 Jena, Germany; 2 Department of Biochemistry and Physiology of Nutrition, Institute of Nutritional Science, Friedrich Schiller University, 07743 Jena, Germany; 3 Competence Cluster for Nutrition and Cardiovascular Health (nutriCARD) Halle-Jena-Leipzig, 07743 Jena, Germany; 4 Department of Pharmaceutical/Medicinal Chemistry, Institute of Pharmacy, Friedrich Schiller University, 07743 Jena, Germany; 5 Jena Center for Soft Matter (JCSM), Friedrich Schiller University, 07743 Jena, Germany

**Keywords:** atopic dermatitis mouse model, 2,4-dinitrochlorobenzene, mild and moderate atopic dermatitis, sub-acute and chronic inflammation

## Abstract

The mouse model of 2,4-dinitrochlorbenzene (DNCB)-induced human-like atopic dermatitis (hlAD) has been widely used to test novel treatment strategies and compounds. However, the study designs and methods are highly diverse, presenting different hlAD disease patterns that occur after sensitization and repeated challenge with DNCB on dorsal skin. In addition, there is a lack of information about the progression of the disease during the experiment and the achieved pheno- and endotypes, especially at the timepoint when therapeutic treatment is initiated. We here examine hlAD in a DNCB-induced BALB/cJRj model at different timepoints: (i) before starting treatment with dexamethasone, representing a standard drug control (day 12) and (ii) at the end of the experiment (day 22). Both timepoints display typical AD-associated characteristics: skin thickening, spongiosis, hyper- and parakeratosis, altered cytokine and gene expression, increased lipid mediator formation, barrier protein and antimicrobial peptide abnormalities, as well as lymphoid organ hypertrophy. Increased mast cell infiltration into the skin and elevated immunoglobulin E plasma concentrations indicate a type I allergy response. The DNCB-treated skin showed an extrinsic moderate sub-acute hlAD lesion at day 12 and an extrinsic mild sub-acute to chronic pheno- and endotype at day 22 with a dominating Th2 response. A dependency of the filaggrin formation and expression in correlation to the disease severity in the DNCB-treated skin was found. In conclusion, our study reveals a detailed classification of a hlAD at two timepoints with different inflammatory skin conditions and pheno- and endotypes, thereby providing a better understanding of the DNCB-induced hlAD model in BALB/cJRj mice.

**Supplementary Information:**

The online version contains supplementary material available at 10.1007/s10753-023-01943-x.

## INTRODUCTION

Atopic dermatitis (AD) is one of the most common skin diseases affecting children with an overall prevalence of 15.5% [1] and up to 3% in adults [2]. Moreover, AD belongs to the type I hypersensitivity reactions, such as allergic asthma or food allergies [3, 4]. The incidence of AD steadily increases and carries a high burden for patients. Co-morbidities such as depression or social anxiety may also likely impair the life quality of AD patients [5–7]. The pathophysiological mechanisms of the disease are highly complex, showing several clinical subtypes with different immune responses and significant variations between ethnic groups [8, 9]. Therefore, expanding the range of therapeutic options focusing on personalized medicine to target specific inflammatory pathways is crucial for future AD treatment [10].

AD mouse models are still indispensable for studying the utility of novel therapeutic compounds. Most popular mouse models are hapten-induced, where topical applied haptens bind to extra- and intracellular proteins [11] leading to inflammatory responses in the skin and lymphoid organs [12, 13]. Repeated treatment of the murine skin with the hapten 2,4-dinitrochlorobenzol (DNCB) induces human-like AD (hlAD) in BALB/c mice [14–23]. Although many studies are using this model, the reproducibility is challenging. Different experimental setups, such as variations in the DNCB concentration and volume, application frequency, or time regimes, strongly hamper the comparability between these studies. Moreover, there is a lack of vital information on DNCB purity and methodology, such as on handling, housing, and hygienic conditions of the mice, size of the treated skin area, or scoring parameters.

The induction of hlAD in the murine skin using DNCB is predominantly described in two phases: a sensitization phase with 1–2% DNCB (first contact with the hapten) once or twice per week and a challenge phase with 0.2–0.5% DNCB (second hapten encounter) for several weeks [14–23]. Although these studies showed similar disease-associated patterns, such as skin abnormalities, altered cytokine and gene expression, increased mast cell infiltration, or elevated immunoglobulin (Ig) E plasma levels, the literature is lacking in information about the achieved AD pheno- and endotypes at the end of the experiment as well as during the course of the experiment. Some studies report a decrease in the severity degree during the experiment [14, 24–28], while other studies seem to maintain or increase disease severity during the experiment [20, 29–32] or only show the endpoint scoring [21, 33–35]. In the literature, there is no identifiable correlating parameter described that is responsible either for the maintenance, reduction, or increase of the disease severity until the end of the experiment. Moreover, only a few studies classified the pheno- or endotype of the DNCB-treated murine skin eczema regarding human AD [27]. Above all, studies are lacking descriptions of the disease severity before starting the therapeutic treatment. Since the choice of the therapeutic agent in managing human AD correlates with the disease severity [36, 37], classification of disease severity and pheno- and endotypes in animal models at this timepoint is essential. Otherwise, the validity of the studies on AD pathogenesis or the evaluation of therapeutic interventions in these models is limited.

We, therefore, developed a DNCB-induced hlAD mouse model and characterized the inflammatory skin conditions (i) at the timepoint before starting the therapeutic treatment with dexamethasone (DEX) at day 12 and (ii) at the end of the experiment at day 22. Our work aims to contribute to a better understanding of how DNCB affects inflammation in the skin during the onset and the progression of AD. In addition, our study intends to enable the selection of an appropriate DNCB regime according to the desired severity of AD diseases to facilitate the assessment of the therapeutic benefit of test substances.

## RESULTS

### DNCB Treatment Regims Cause Different Severities of Atopic Dermatitis

**Fig. 1:**
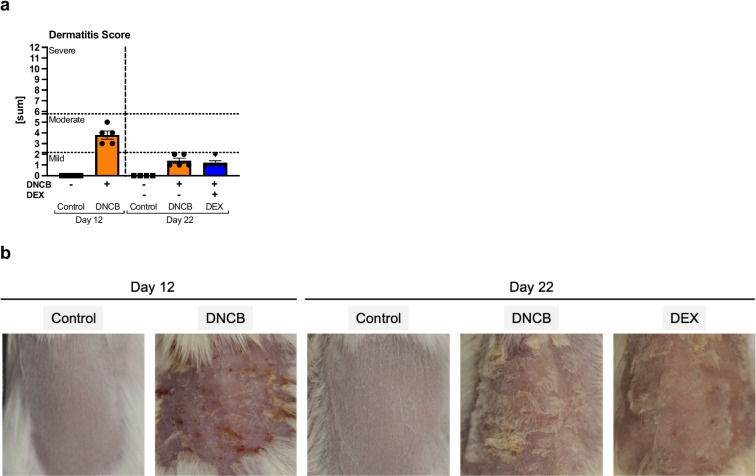
DNCB-induced hlAD in dorsal murine skin shows different severity degrees during the experiment.** a** The Dermatitis Score represents the evaluation of four summarized symptoms (skin dryness, erythema/redness, edema, and excoriation/crust formation). The dotted lines indicate the cut-off values of mild, moderate, or severe severity of AD. **b** Representative images of the treated skin area at day 12 and day 22. Data are presented as means ± standard error of the mean (SEM; *n* = 4–5 per group). Squares, dots, and rhombi represent individuals.

hlAD was induced on the dorsal skin of BALB/cJRj mice with 1% DNCB in the sensitization phase and repeated challenge with 0.3% DNCB in the second phase. Different degrees of skin inflammation were observed before starting the DEX treatment (day 12) and at the end of the experiment (day 22) (Fig. 1a, b). At day 12, the DNCB-treated skin showed the highest hlAD eczema severity, with a Dermatitis Score of 3.8 ± 0.4 indicating a moderate disease severity. At day 22, a mild disease expression was observed (Dermatitis Score: 1.4 ± 0.2 for DNCB vs. 1.2 ± 0.2 for DEX). The control groups did not show skin inflammation.

## SKIN TREATMENT WITH DNCB INDUCES HYPERTROPHY OF SECONDARY LYMPHOID ORGANS

**Fig. 2:**
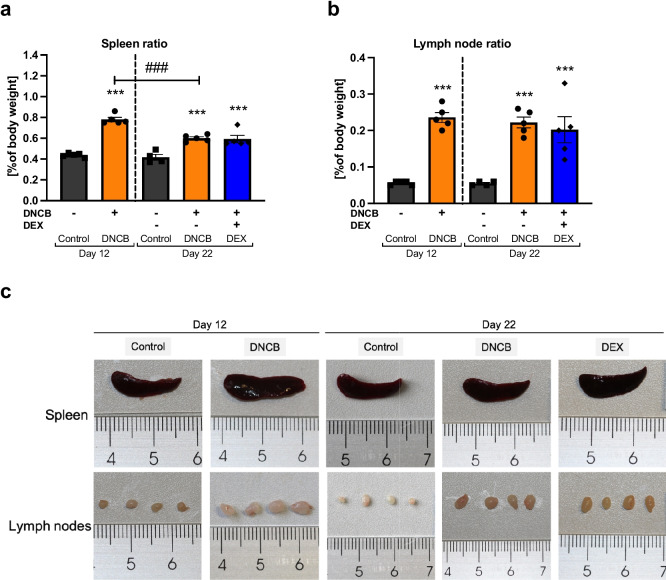
DNCB-treatment on murine dorsal skin induces secondary lymphoid organ hypertrophy.** a** Spleen ratio. **b** Lymph node ratio. **c** Representative images of the respective spleen and lymph nodes at day 12 and day 22. Data are presented as means ± SEM (*n* = 4–5 per group). Squares, dots, and rhombi represent individuals. ^***^p < 0.001, comparison versus respective time control. ^###^*p* < 0.001, comparison between the DNCB groups.

One essential role of peripheral lymphoid organs, such as lymph nodes and spleen, is to retain lymphocytes. In response to antigens, these immune cells are activated, proliferate, and mature [38, 39], which plays a pivotal role in the pathogenesis of AD. Consequently, the lymphoid organs enlarge. In our study, mice from all DNCB groups, as well as the DEX group showed significantly increased spleen weight and size compared to the respective controls at both timepoints (Fig. 2a; ^***^p < 0.001; Fig. 2c). The spleen ratio decreased significantly during the experiment in the DNCB group (Fig. 2a; ^###^*p* < 0.001). Abdominal and axillary lymph nodes were also significantly increased in size and weight in both DNCB groups as well as in the DEX group (Fig. 2b; ^***^p < 0.001; Fig. 2c). However, no differences were detected between the two DNCB groups from day 12 to day 22 (Fig. 2b). DEX treatment did not influence the secondary lymphoid organ ratio compared to the DNCB group (Fig. 2a, b).

## DNCB TREATMENT CHANGES MORPHOLOGICAL SKIN STRUCTURE

**Fig. 3:**
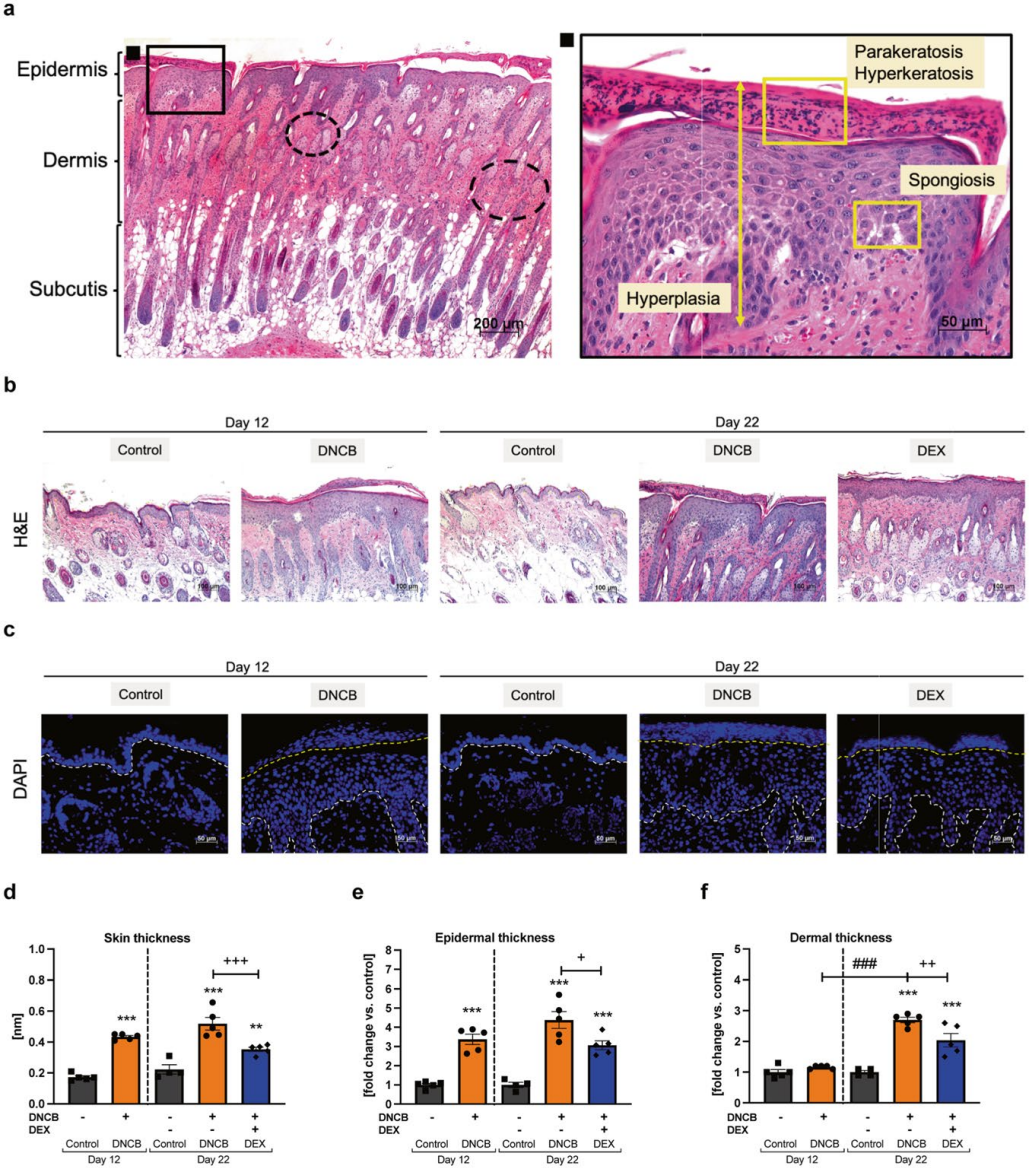
DNCB-treated dorsal skin shows hlAD skin characteristics.** a** Representative hematoxylin and eosin (H&E) staining of DNCB-treated dorsal skin. Skin sections show increased epidermis and dermis with subepidermal sclerosis (dashed circles) and subcutis at day 22. Scale bar 200 µm. Enlarged image (∎) of acanthotic skin area demonstrating the thickening of the epidermis (hyperplasia), hyperkeratosis, parakeratosis, and spongiosis. Scale bar 50 µm. **b** Representative H&E-stained sections of tissues of each group. Scale bar: 100 µm. **c** Respective 4′,6-diamidin-2-phenylindol (DAPI)-stained sections of each group showing cell nuclei. White dashed lines separate the dermis from epidermis. Area above the yellow dashed lines shows cell nuclei in the stratum corneum of the DNCB-treated skin. Scale bar: 50 µm. **d** Evaluation of the skin thickness. **e** Evaluation of the epidermal and **f** dermal thickness on H&E-stained sections compared to the respective control. Data are presented as means ± SEM (*n* = 4–5 per group). Squares, dots, and rhombi represent individuals. ^*^p < 0.05, ^**^p < 0.01, and ^***^p < 0.001, comparison versus respective time control or ^+^*p* < 0.05, ^++^*p* < 0.01, and ^+++^*p* < 0.001 DEX treatment versus DNCB control. ^###^*p* < 0.001, comparison between the DNCB groups.

Hematoxylin and eosin (H&E) staining of dorsal sections (Fig. 3a, b) revealed hyperplasia, hyperkeratosis, parakeratosis, and spongiosis in the DNCB-treated skin. Increased proliferation of epidermal keratinocytes in the DNCB-treated skins was found by fluorescence staining of the cell nuclei, also indicating retention of nuclei in the stratum corneum (Fig. 3c). Consequently, significant changes in total skin thickness and epidermal thickness of the DNCB-treated skin areas compared to the respective time control were confirmed at both timepoints (Fig. 3d, ^**^p < 0.01, ^***^p < 0.001; Fig. 3e, ^***^p < 0.001). No differences were detected between the DNCB groups at the different timepoints. DEX treatment significantly reduced total skin and epidermal thickness (Fig. 3d, ^+++^*p* < 0.001; Fig. 3e, ^+^*p* < 0.05). Dermal thickness increased significantly only after DNCB treatment at day 22 (Fig. 3f, ^***^p < 0.001, ^###^*p* < 0.001), which was found to be distinctly decreased by treatment with DEX (Fig. 3f, ^++^*p* < 0.01).

## DNCB TREATMENT INDUCES A TYPE I ALLERGIC RESPONSE

**Fig. 4:**
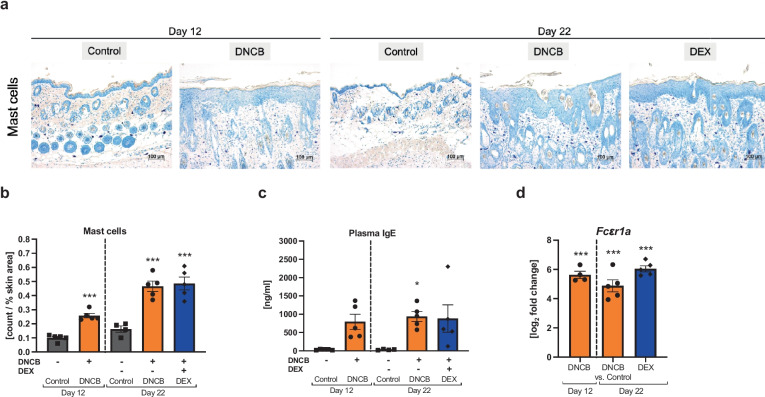
DNCB treatment of dorsal murine skin indicates a type I allergy response reaction.** a** Representative images of toluidine-stained sections. Scale bar: 100 µm. **b** Evaluation of mast cell infiltration on toluidine-stained sections. **c** IgE concentrations in plasma. **d** Relative mRNA expression of *Fc* $\varepsilon$ *R1a* in the skin. Data are presented as means ± SEM (*n* = 4–5 per group). Squares, dots, and rhombi represent individuals. ^*^p < 0.05 and ^***^p < 0.001, comparison versus respective time control.

AD is also known as a type I hypersensitivity reaction, an immediate type I reaction mediated by cross-linking of the high-affinity FC $\upvarepsilon$ R1A receptor on mast cells or basophils via IgE [40, 41]. Elevated mast cell infiltration into the skin (Fig. 4a) was found in the DNCB-treated animals compared to controls at both timepoints (Fig. 4b, ^***^p < 0.001). Simultaneously, IgE concentrations in the plasma increased in the DNCB group at day 22 (Fig. 4c, ^*^p < 0.05). A similar trend was observed at day 12 in the DNCB group (Fig. 4c, p = 0.06) and in the DEX group at day 22 (Fig. c, *p* = 0.06). Treatment with DEX neither affected the allergic response compared to the respective DNCB group at day 22, nor did the DNCB groups show differences between the two timepoints. Significantly increased mRNA expression of *Fc* $\varepsilon$ *r1a* was found at both timepoints in the DNCB groups, whereas treatment with DEX did not influence the DNCB-induced expression (Fig. d, ^***^p < 0.001).

## DNCB ALTERS SKIN BARRIER PROTEINS AND ANTIMICROBIAL PEPTIDES

**Fig. 5:**
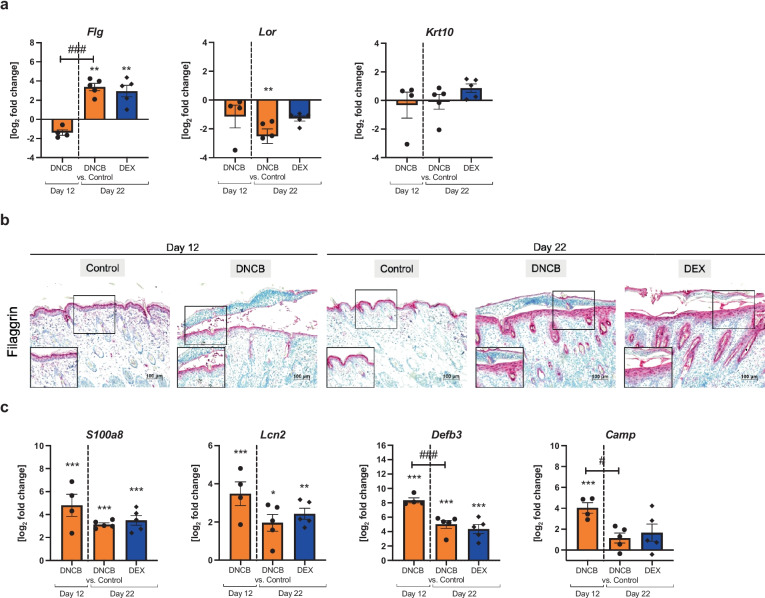
DNCB treatment of dorsal murine skin influences skin barrier proteins and expression of antimicrobial peptides.** a** Relative mRNA expression of skin barrier proteins. **b** Representative images of the filaggrin-stained sections of all groups. Scale bar: 100 µm. **c** Relative mRNA expression of antimicrobial peptides in the skin. Data are presented as means ± SEM (*n* = 4–5 per group). Dots and rhombi represent individuals. ^*^p < 0.05 and ^**^p < 0.01, comparison versus respective time control. ^#^*p* < 0.05 and ^###^*p* < 0.001, comparison between the DNCB groups.

Skin barrier defects are one of the main characteristics of AD, where the barrier proteins filaggrin, loricrin, and cytokeratin-10 play a pivotal role in skin integrity. Relative mRNA expression of the gene encoding filaggrin (*Flg*) was downregulated at day 12 in the DNCB-treated skin and upregulated at day 22 (Fig. 5a, ^**^p < 0.01, ^###^*p* < 0.001). In line with this, filaggrin was reduced in immunochemical stained slides, visible as diminished staining intensity per cell, on day 12 compared to day 22 (Fig. 5b). Another skin barrier protein loricrin (*Lor*) was downregulated at both timepoints with a significant effect at day 22 in the DNCB-treated skin (Fig. 5a, ^**^p < 0.01), while cytokeratin-10 (*Krt10*) was not markedly altered in all groups (Fig. 5a). DEX treatment did not alter gene expression of the skin barrier proteins compared to the DNCB group (Fig. 5a).

Skin is primarily protected by antimicrobial peptides (AMPs) against pathogens and AMPs are also involved in AD pathogenesis. Relative mRNA expression of the S100-calcium binding protein a8 (*S100a8*), lipocalin-2 (*Lcn2*), and β-defensin 3 (*Defb3*) was significantly upregulated in the DNCB-treated skin at both timepoints (Fig. 5c, ^*^p < 0.05, ^***^p < 0.001) and of the cathelicidin antimicrobial peptide (*Camp*) at day 12 (Fig. 5c, ^***^p < 0.001). All AMPs tend to be less upregulated at day 22 compared to day 12, with a significant difference detectable for *Defb3* and *Camp* (Fig. 5c, ^#^*p* < 0.05, ^###^*p* < 0.001). DEX treatment did not influence the gene expression compared to the respective DNCB group.

## Th2/Th1 CYTOKINE PATTERN IS MODULATED BY PROLONGED DNCB TREATMENT

**Fig. 6:**
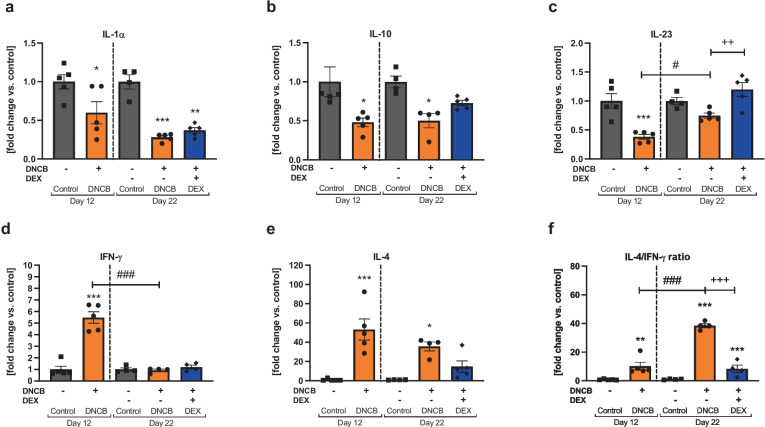
DNCB treatment alters cytokine expression compared to the healthy control in the dorsal murine skin.** a, b** Pro- and anti-inflammatory cytokine expressions in the skin. **c–f** T helper cell-related cytokine expression in the skin. Data are presented as means ± SEM (*n* = 4–5 per group). Squares, dots, and rhombi represent individuals. ^*^p < 0.05, ^**^p < 0.01, and ^***^p < 0.001, comparison versus respective time control or ^++^*p* < 0.01 DEX treatment versus DNCB control. ^#^*p* < 0.05 and ^###^*p* > 0.001, comparison between the DNCB groups.

The acute phase pro-inflammatory interleukin (IL)-1α as well as the anti-inflammatory cytokine IL-10 were decreased in DNCB-treated skin areas compared to the respective controls (Fig. 6a, b, ^*^p < 0.05, ^**^p < 0.01, ^***^p < 0.001). The pro-inflammatory cytokine IL-23 was significantly reduced in the DNCB group compared to the control group at day 12, whereas a significant increase was observed between the DNCB-treated group at day 22 compared to day 12 (Fig. 6c, ^***^p < 0.001, ^#^*p* < 0.05). DEX treatment fully reversed IL-23 secretion in the DNCB-treated skin compared to the healthy skin level (Fig. 6c, ^++^*p* < 0.01). Interferon (IFN)-γ, a T helper type-1 (Th1) cell-mediated cytokine, was increased at day 12 in the DNCB group (Fig. 6d, ^***^p < 0.001), while the expression decreased to a normal level after day 22 (Fig. 6d, ^###^*p* < 0.001). Analysis of IL-4, a T helper type-2 (Th2) cell-mediated cytokine, showed significantly elevated levels in the DNCB-treated skin at both timepoints, with a more significantly pronounced effect at day 12 (Fig. 6e, ^*^p < 0.05, ^***^p < 0.001). It is known that the immune response pattern of Th2/Th1 cells shifts during the transition from an acute to a chronic stage in human AD [42]. Evaluation of this shift, represented by the IL-4/IFN-$\gamma$ ratio, depicts a clear enhancement of the Th2 immune response compared to the Th1 immune response at the end of the experiment (Fig. 6f, ^###^*p* < 0.001). Treatment with DEX reduced this ratio to an equal level as detected on day 12 (Fig. 6f,  ^+++^*p* < 0.001).

## DNCB TREATMENT INFLUENCES PRO-AND ANTI-INFLAMMATORY LIPID MEDIATOR FORMATION

**Fig. 7:**
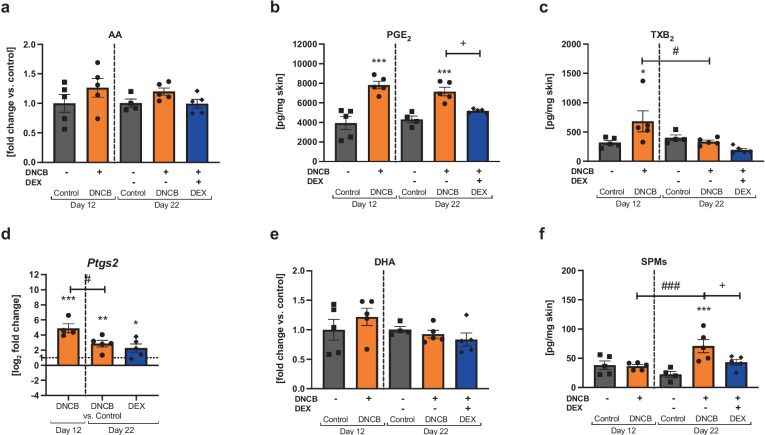
DNCB treatment of dorsal murine skin alters the formation of pro- and anti-inflammatory lipid mediators as well as specialized pro-resolving mediators compared to the healthy control in the dorsal murine skin.** a** Arachidonic acid release (AA). **b** Prostaglandin (PG) E_2_ and **c** thromboxane (TX) B_2_ formation. **d** Relative mRNA expression of *Ptgs2* in the skin. **e** Release of docosahexanoic acid (DHA) in the skin. **f** Total sum of specialized pro-resolving lipid mediators (SPMs) in the skin (resolvin D_5_, protectin DX). Data are presented as means ± SEM (*n* = 4–5 per group). Squares, dots, and rhombi represent individuals. ^*^p < 0.05, ^**^p < 0.01, and ^***^p < 0.001, comparison versus respective time control or ^+^*p* < 0.05 DEX treatment versus DNCB control. ^#^*p* < 0.05 and ^##^*p* > 0.01, comparison between the DNCB groups.

The inflammatory skin milieu was assessed for the presence of inflammation-related lipid mediators (LMs). Pro-inflammatory LMs are derived from polyunsaturated fatty acids, such as arachidonic acid (AA) [43]. AA release was not significantly altered in all groups (Fig. 7a). Prostaglandin (PG) E_2_ was the most prominent pro-inflammatory LM in the DNCB-treated skin at both timepoints, while DEX treatment blocked this formation to an equal level of the respective healthy control (Fig. 7b, ^***^p < 0.001, ^+^*p* < 0.05). The thromboxane (TX) B_2_ was only significantly increased at day 12 in the DNCB group and already decreased at day 22 (Fig. 7c, ^*^p < 0.05, ^#^*p* < 0.05). PGs and TXs are biosynthesized by cyclooxygenases (*PTGS*) from AA [43]. While the level of mRNA expression of *Ptgs2* was elevated in the DNCB-treated skin at both timepoints compared to the controls (Fig. 7c, ^**^p < 0.01, ^***^p < 0.001), the expression was significantly decreased at day 22 compared to day 12 (Fig. 7d, ^#^*p* < 0.05). No effect of the DEX treatment was noted at the transcription level compared to the respective DNCB group (Fig. 7d). Besides pro-inflammatory LM formation, we also investigated the appearance of docosahexaenoic acid (DHA)-derived specialized pro-resolving mediators (SPMs), which fulfill several functions in the resolution process of inflammation [44]. The DHA concentration was not significantly elevated in all groups (Fig. 7e), while SPMs were significantly increased at day 22 in the DNCB group compared to the respective control and to day 12 (Fig. 7d, ^***^p < 0.001, ^###^*p* < 0.001). Treatment with DEX inhibited the DNCB-induced SPM formation to a level compared to the control (Fig. 7d, ^+^*p* < 0.05). LM profiles assessed by metabololipidomics are shown in Fig. S1.

## DISCUSSION

In our study, hlAD was induced in mice using 1% DNCB in the sensitization phase, followed by repeated challenge with 0.3% DNCB. To investigate local and systemic inflammation during the course of the experiment, healthy and DNCB-treated animals were evaluated in regard to several disease parameters at days 12 and 22. The respective timepoints were chosen for the following reasons: (i) therapeutic treatment, using DEX as a standard drug control in this model, started at day 12, and (ii) day 22, which defines the end of the experiment. To our knowledge, there is no official guideline for evaluating the severity of AD in murine skin that is similar to clinical tools used of human AD such as the SCORing Atopic Dermatitis (SCORAD) score [45]. This hampers the comparability of the hlAD manifested in our model to other published studies. Nevertheless, we scored two different disease severities during the experiment, which are similar to moderate (day 12) and mild (day 22) human AD.

A Th cell imbalance is a main characteristic of AD pathogenesis [42, 46–48]. Lymph nodes store unmatured naïve Th cells which mature and polarize after contact with hapten-loaded antigen-presenting cells emigrated from the skin [38, 49, 50]. Hypertrophy of lymphoid organs after DNCB application to the skin was shown in the literature [15, 19, 21, 24, 51]. In our study, lymph nodes and spleen were morphologically altered at both timepoints. However, the spleen seems to be more sensitive to changes in the inflammatory conditions since the hypertrophy was reduced from day 12 to day 22.

Thickening of the skin is a disease hallmark of the DNCB-induced hlAD skin [52–56]. This effect was also shown in skin hyperplasia and epidermal thickening at both timepoints in our study, but dermal thickening was only detectable at the end of the experiment. As described in the literature and shown in our study, DEX treatment regulates this effect in the epidermal and dermal layers [52, 54, 55]. Epidermal thickening occurs in mild, moderate, and severe lesions of AD patients [48], as well as in acute and chronic lesions [42]. However, alteration of the dermal structure (cutaneous fibrosis) is often a symptom of chronic inflammation leading to the accumulation of extracellular matrix [57]. Subepidermal sclerosis was only measured in the mild hlAD, with a transition to a chronic inflammatory skin milieu being assumed from day 12 to day 22. The presence of spongiosis (epithelial intercellular edema) is more associated with acute to sub-acute lesions and minor to absent in chronic AD lesions, which are accompanied by irregular acanthosis, hyperkeratosis, and parakeratosis [42, 47, 58, 59]. We found spongiosis at both timepoints. According to our histological results, we classify a sub-acute AD phenotype at day 12 and a sub-acute to chronic AD phenotype, mainly indicated by the cutaneous fibrosis found at the end of the experiment (day 22).

During epidermal differentiation, keratinocytes originating from the basement membrane proliferate and differentiate, while gene expression switches from early to late differentiation markers. We found differing mRNA levels of the late differentiation marker and skin barrier protein *Flg* in the DNCB-induced hlAD skin*,* depending on the disease severity. While *Flg* was downregulated in the moderate hlAD lesions at day 12, the expression was upregulated at the end of the experiment, indicating mild hlAD. This finding is in line with the results from the literature, where *Flg* downregulation is described for DNCB-induced moderate to severe hlAD [21, 60, 61] but not for mild hlAD [27] or mild human AD [62]. *Krt10,* an early differentiation marker, remained unchanged by DNCB treatment in our study. DEX treatment did not influence the gene expression.

AD belongs to the group of “atopic” diseases, which show type I hypersensitive responses to antigens by IgE-mediated mast cell degranulation via cross-linking of the FC $\upvarepsilon$ R1A receptor and release of inflammatory mediators [40]. An intrinsic and extrinsic form is distinguished in human AD, with the latter being accompanied by high serum IgE level [41, 62]. In our study, the allergic response, determined by elevated IgE levels in the plasma, increased mast cell infiltration into skin, and upregulated *Fc* $\varepsilon$ *R1a* mRNA expression, did not differ between the timepoints or disease severity. Since the half-life of IgE in serum is very short in mice (12 h) [63, 64], we interpret our findings as an ongoing and stable type I allergy response to DNCB until the end of the experiment.

IgE synthesis is regulated by cytokines expressed by polarized Th2 cells after antigen contact. It was shown that the Th2 cytokine IL-4 induces the class switch of IgE production in B cells, while the Th1 cell-mediated cytokine IFN-γ inhibits this effect [65, 66]. IL-4 was found to be upregulated in the DNCB-treated groups in our study, mirroring this pathomechanistic context. A dominant Th2 immune response is related to acute AD lesions in humans [42, 67], while chronic lesions involve Th1 responses [42, 46, 68]. Moreover, IFN-γ is known for skin-thickening effects in murine skin [69]. We expected an increased IFN-γ level at day 22. However, IFN-γ was only significantly increased at day 12. Consequently, the Th2/Th1 cell-mediated cytokine ratio was markedly elevated until day 22. This is in line with the findings by Kitagaki et al. [70], who detected a shift from a Th1 immune response in acute lesions to Th2 immune response in chronic lesions after repeated antigen elicitation. Taken together, in our study, the DNCB treatment induced chronic lesion in the murine skin of BALB/cJRj mice with a dominating Th2 immune response at the end of the experiment.

The pro-inflammatory cytokine IL-23 plays a central role in the pathogenesis of psoriasis, another common chronic skin disorder [71]. Reduced IL-23 expression was found in chronic AD lesions [72]. The decreased IL-23 cytokine levels in both DNCB-treated groups indicate a lack of the IL-23/Th17 signaling pathway and distinguish the disease-associated skin in this model from human-like psoriatic skin.

The role of AMPs in AD is discussed controversially in the literature. However, progressive evidence is promoting an AMP induction in AD [73]. In our study, mRNA levels of the analyzed AMPs were upregulated in the DNCB-induced hlAD skin. This was also shown in human skin biopsies from chronic lesions of extrinsic AD [74].

Furthermore, the inflammatory skin milieu was investigated by metabololipidomics of polyunsaturated fatty acids and LM formation. PGs and TXs are LMs produced by the cyclooxygenase pathway [43] and the coding gene *Ptgs2* for cyclooxygenase-2 was upregulated on the mRNA level at both timepoints in relation to the disease severity. Previously, PGE_2_ was shown to be the mainly generated pro-inflammatory LM in DNCB-induced mild hlAD [27]. This was also found in our study; however, the effect was not dependent on the disease severity. DEX treatment inhibited PGE_2_ formation at day 22. This effect is described for anti-inflammatory steroids in several tissues in literature [75]. TXB_2_, which is a measurable and a stable metabolite of TXA_2_, was only upregulated at day 12. It is assumed that TXA_2_ is involved in itching in murine skin [76]. A recent study observed a moderate correlation between the itch severity and the AD severity of patients [77], which may be similar for hlAD murine skin. SPMs play an essential role in resolving the inflammatory response [78]. The SPMs resolvin D_5_ and protectin DX were increased at day 22 in the DNCB-induced hlAD skin, which was just demonstrated in mild hlAD [27].

To summarize, our DNCB-induced hlAD model depicts two different stages of disease severity during the course of the experiment. At day 12, we found a moderate extrinsic hlAD pheno- and endotypei, which transformed into a mild extrinsic hlAD skin lesion at day 22. At both timepoints, we found basic criteria that confirm AD-typical local skin inflammation and abnormalities. To our knowledge, we are the first to show a correlation between filaggrin expression and disease severity in the DNCB-induced hlAD skin of BALB/cJRj mice. Maintenance of the filaggrin deficiency might be achieved in this model through higher DNCB concentrations in the challenge phase. We consider this as an important finding for scientists intending to work with this model with a focus on the effect of their therapeutic compounds on filaggrin. Moreover, we provide more information on the scarcely described LM formation in the DNCB-induced hlAD skin. We further detected a mixture of a sub-acute phenotype in the moderate AD and sub-acute to chronic Th2-like skin lesions (marked epidermal thickening, fibrosis, increased ratio of Th2/Th1 immune response) in the mild hlAD. However, more data are needed to definitely classify the hlAD pheno- and endotypes in the DNCB-induced hlAD. Nevertheless, it is important to note that none of the models fully reflect the complexity of human AD but rather partial aspects of the disease [79]. In conclusion, our study provides a better understanding of the DNCB-treated murine skin in regard to human AD and thus, to the evaluation of this model for treatment strategies of novel medications in the therapy of AD.

## MATERIAL AND METHODS

### Chemicals

DNCB (Cat. No. 237329), olive oil (Cat. No. 75343), phosphate-buffered saline (PBS) (Cat. No. D8537), and dexamethasone (DEX) VETRANAL® (Cat. No. 46165) were purchased from Sigma-Aldrich (Darmstadt, Germany) and acetone from VWR international (Darmstadt, Germany) (Cat. No. 270725). DNCB was dissolved in acetone and olive oil (3:1, v/v). Dimethyl sulfoxide (DMSO) was purchased from Carl Roth (Cat. No. A994.2; Karlsruhe, Germany).

## ANIMALS AND HOUSING CONDITIONS

Specific-opportunistic pathogen-free (SOPF) BALB/cJRj (8-week-old, female; JanvierLabs, Saint Berthevin Cedex, France) were acclimatized for 1 week as described [27] under SOPF conditions in accordance to the Federation of European Laboratory Animal Science Associations (FELASA) recommendations [80]. Testing of pathogens was conducted in a 3-month frequency. All animals were kept as two to three individuals in individually ventilated cages (Green Line 500, TecniPlast, Germany) with stable housing conditions (temperature, 22 ± 2 °C; humidity, 55 ± 10%; 12:12-h light–dark cycle) and access to a standard rodent diet (Ssniff Spezialdiäten; Cat. No. V1534-300, Soest, Germany) and autoclaved water *ad libitum*. Each cage was enriched with a cardboard tunnel, poplar wood pellets, cotton rolls as nesting material, and a wooden stick. Cages and freshwater were changed once a week.

## INDUCTION OF ATOPIC DERMATITIS

**Fig. 8:**
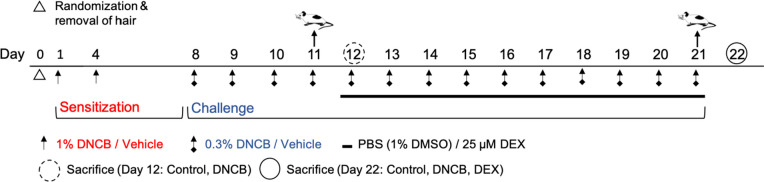
Time regime of the DNCB-induced hlAD model using BALB/cJRj mice. DNCB sensitization and challenge or treatment with vehicle (aceton/olive oil, 3:1, v/v) were applied topically on the dorsal skin and behind the right ear. PBS (with 1% DMSO) or dexamethasone (DEX) were applied daily on the dorsal area, 1 h after DNCB or vehicle treatment, from day 12 to day 21. Animals were taken out of the experiment at day 11 and day 21 and sacrificed on the following day (day 12 and day 22).

After acclimatization, the animals were anesthetized with isoflurane, and a 5 cm^2^ area of the back of each mice was shaved (Aesculap Exacta GT416, B. Braun Melsungen, Melsungen, Germany) and hair routs were removed by depilation cream (Veet, Reckitt Benckiser, Heidelberg, Germany; Cat. No. (PZN) 7,768,307). The animals were randomized and divided into five groups: Control at day 12 (*n* = 5); DNCB at day 12 (*n* = 5); control at day 22 (*n* = 4); DNCB at day 22 (*n* = 5); DEX at day 22 (*n* = 5). After one day of recovery, the animals from the DNCB groups and the DEX group were sensitized by pipetting 200 µl of 1% DNCB on the depilated dorsal skin and 20 µl of 1% DNCB behind the right ear (Fig. 8). The DNCB treatment was repeated at day 4. From day 8 on, the animals were treated daily with 0.3% DNCB until day 21. The control groups were treated with the vehicle (acetone to olive oil, 3:1, v/v). For application, the animals were fixed on the root of their tail. After the DNCB-induction on day 11, the control and the DNCB group were taken out of the experiment for characterization of the inflammatory skin conditions on the following day (day 12). From day 12, the control and the DNCB group were treated with PBS (with 1% DMSO) and the DEX group with 25 µM DEX (PBS with 1% DMSO) approximately 1 h after DNCB or vehicle treatment by slowly applying 150 µl to the treated skin and gently spreading with a soft brush. This procedure was documented by video recording (Supplementary file 2 FinePix XP130; Fujifilm Corporation, Tokyo, Japan). Animals were sacrificed on the following day. All animals (day 12 and day 22) were anesthetized with ketamine (100 mg/kg/body weight) und xylazine (15 mg/kg/body weight) and blood was taken from the heart with syringes containing 0.5 M ethylendiaminetetraacetic (EDTA).

The skin, spleen, and lymph nodes were weighed and photographed (Canon EOS 600D; Canon, Krefeld, Germany) and stored overnight at 4 °C in PBS. Brightness of spleen and lymph node images was equally adjusted using PowerPoint™ Microsoft 365 (Redmond, WA, USA). Skin thickness of the treated area was measured using a digital measuring stick (IP67, 150 mm, Mitutoyo, Japan). Skin samples for gene expression analysis were immediately covered with RNA tissue protect reagent (Qiagen, Hilden, Germany) and stored at − 80 °C. Skin samples for LM analysis and cytokine expression were weighed and directly stored at − 80 °C.

## SCORING

Body weight was measured weekly. Dermatitis Score was calculated based on four disease-specific symptoms: dryness, erythema and redness, edema, and excoriation/crust formation as described [27]. Each symptom was scored with 0 (none), 1 (mild), 2 (moderate), or 3 (severe). The evaluation of the disease severity was conducted in accordance with the objective SCOring of Atopic Dermatitis (oSCORAD) score [37, 81] at day 12 and after a second shave at day 22. The oSCORAD classifies the mild eczema with a score less than 15 (18.1%), the moderate eczema from 15 to 40 (48.2%) and the severe eczema greater than 40 with a maximum score of 83. In our study, the dermatitis score was designed as the sum of each individual symptom with a maximum score of 12. By calculation of the percentage cutoff values from the oSCORAD score, the cutoff values in our study were calculated by the transfer of these percentages to the total dermatitis score of 12. The cutoff values were defined as follows: Mild: < 2.2 (< 18.1%); moderate: 2.2–5.8 (18.1–48.2%); severe: > 5.8 (> 48.2%). Photographs were taken using a digital camera (Canon EOS 600D). The brightness of images was equally adjusted using PowerPoint Microsoft 365 (Microsoft).

## MEASUREMENT OF IGE CONCENTRATION IN PLASMA

EDTA-blood was stored for 30 min at room temperature (RT), centrifuged (300 × g, 10 min, RT), and plasma was aliquoted and stored at –80 °C. IgE was measured using a fluorescence-encoded bead-based assay on flow cytometry according to the manufacturer’s protocol (BioLegend, Amsterdam, Netherlands).

## CYTOKINE MEASUREMENT IN THE SKIN

The preparation of 100 mg skin tissue was conducted as described previously [27]. The cytokines in the supernatant were measured with a fluorescence-encoded bead-based assay using flow cytometry according to the manufacturer’s protocol (LegendPlex, BioLegend).

## HISTOLOGICAL AND IMMUNOCHEMICAL STAINING

The right ear and 6 mm of the dorsal skin were fixed with 4% phosphate-buffered formaldehyde (Carl Roth) at room temperature for 24 h and then stored in PBS until embedding in paraffin blocks. Further preparation and staining was recently described [27]. Samples were sliced as 4 µm sections and dewaxed, followed by heat-induced epitope retrieval. Slides were blocked with UltraCruz Blocking Reagent (Santa Cruz Biotechnology, Dallas, TX, USA) and the Avidin/Biotin Blocking System (BioLegend). Immunohistological staining of filaggrin was conducted with a primary rabbit-anti-mouse antibody (1:2000, MyBioSource, San Diego, CA, USA) and a secondary biotin-labed antibody (Jacksin ImmunoResearch, Ely, UK). After incubation with VECTASTAIN ABC-AP Kit (Vector Laboratories, Newark, CA, USA), samples were treated with an alkaline phosphatase substrate (Abcam, Cambridge, MA, USA), counterstained with hematoxylin Gill I (Merck), and covered with a coverslip plus aquatex (Merck). H&E staining was performed with Haematoxylin Grill II (Merck) and Eosin Y (Merck), and samples were covered with a coverslip plus Histofluid (Paul Marienfeld, Lauda-Königshofen, Germany). Toluidine staining was conducted with 0.1% toluidine blue O (Sigma-Aldrich) in 0.9% sodium chloride before samples were covered with a coverslip and Histofluid (Paul Marienfeld). Cell nuclei were stained with 4,5-diamidino-2-phenylindole (DAPI; Sigma-Aldrich). Photographs of stained slides were taken with the digital camera AxioCam MRc (Carl Zeiss, Jena, Germany), and microscopic assessment was carried out on the Axio Scope A.1 microscope (Carl Zeiss). DAPI filter was set to a wavelength from $\lambda$_ex_ = 358 nm. The brightness of the images was adjusted using PowerPoint Microsoft 365 (Microsoft). Evaluation of epidermal and dermal thickness was conducted with ImageJ version 1.53t [82]. Mast cell infiltration into the skin was counted on toluidine-stained slides using ImageJ and calculated as a ratio to the relative area of the photographed skin.

## RNA ISOLATION, cDNA SYNTHESIS, AND QUANTITATIVE REAL-TIME PCR

RNA of skin samples was isolated and purified with the RNeasy Mini Kit (Qiagen). Approximately 30 mg skin sample was cut into small pieces and homogenized with buffer RLT (Qiagen) and β-mercaptoethanol (Carl Roth) using a tissue ruptor. Disrupted samples were then incubated with proteinase K (ThermoFisher) at 55 °C (1000 rpm, 10 min). After the addition of buffer, RLT samples were centrifuged (10,000 × g, 3 min), and cleared lysate was mixed with 70% ethanol in a fresh tube. The sample was then transferred to an RNeasy spin column and centrifuged (8000 × g, 15 s), while the flow-through was discarded. Buffer RW1 (Qiagen) was then added into the column (8000 × g, 15 s). DNase (Qiagen) digestion was performed by on-column incubation for 15 min at room temperature. Buffer RW1 was further added, and the column was centrifuged (8000 × g, 15 s). The same procedure was conducted with buffer RPE (Qiagen). The spin column was placed in a new tube, and 30 µl RNase-free water was directly added to the spin column membrane and centrifuged (8000 × g, 1 min), which was once repeated with the same eluate. RNA quantification was conducted with a spectrometer (NanoDrop; ThermoFisher). cDNA synthesis was conducted with the RT^2^ First Strand Kit (Qiagen) according to the manufacturer’s protocol. cDNA was added together with RT^2^ SYBR Green Mastermix (Qiagen) into a customized 384-well RT^2^ Profiler PCR Array plate (Qiagen) with a gene panel (Table S1). Real-time PCR was conducted with a LightCycler 480 (Roche Diagnostics, Mannheim, Germany). The cycling conditions were programmed as recommended in the manufacturer’s protocol. Relative mRNA expression was calculated by the 2^−ddCt^-method normalized to peptidylprolyl isomerase H as a housekeeping gene. Fold changes were log_2_ transformed.

## METABOLOLIPIDOMICS PROFILING BY UPLC-MS–MS

The lipid mediators (LMs) were analyzed by UPLC-MS/MS as described by Riedl et al. [27]. In brief, 20 mg of each skin sample was extracted in 400 µl PBS using a FastPrep-24 5G Lysis System (MP Biomedicals, Eschwege, Germany). After the skin samples were extracted, 800 µl of ice-cold methanol containing deuterated LM standards (200 nM d8-5S-HETE, d4-LTB_4_, d5-LXA_4_, d5-RvD2, d4-PGE_2_, and 10 µM d8-AA; Cayman Chemical/Biomol, Hamburg, Germany) was added. Samples containing deuterated LM standards were kept at − 20 °C for at least 60 min to allow protein precipitation. After centrifugation (1200 × g; 4 °C; 10 min) acidified H_2_O (9 mL; final pH = 3.5) was added, and samples were extracted on solid phase cartridges (Sep-Pak Vac 6 cc 500 mg/6 mL C18; Waters, Milford, MA, USA). Samples were loaded on the cartridges after equilibration with methanol followed by water. After washing with water and n-hexane, samples were eluted with methyl formate (6 mL). The solvent was fully evaporated using an evaporation system (TurboVap LV, Biotage, Uppsala, Sweden), and the residue was resuspended in 150 µl methanol/water (1:1, v/v) for UPLC-MS–MS analysis. LMs were analyzed with an Acquity UPLC system (Waters, Milford, MA, USA) and a QTRAP 5500 Mass Spectrometer (ABSciex, Darmstadt, Germany) equipped with a Turbo V Source and electrospray ionization. LMs were eluted using an ACQUITY UPLC BEH C18 column (1.7 µm, 2.1 mm × 100 mm; Waters, Eschborn, Germany) heated at 50 °C with a flow rate of 0.3 mL/min and a mobile phase consisting of methanol–water-acetic acid at a ratio of 42:58:0.01 (v/v/v) that was ramped to 86:14:0.01 (v/v/v) over 12.5 min and then to 98:2:0.01 (v/v/v) for 3 min [83]. The QTRAP 5500 was run in negative ionization mode using scheduled multiple reaction monitoring (MRM) coupled with information-dependent acquisition. The scheduled MRM window was 60 s, and optimized parameters of LMs were adopted, with a curtain gas pressure of 35 psi [83]. The retention time and at least six diagnostic ions for each LM were confirmed by means of an external standard for each and every LM (Cayman Chemical/Biomol, Hamburg, Germany). Quantification was achieved by calibration curves for each LM. Linear calibration curves were obtained for each LM and gave *r*2 values of 0.998 or higher. The limit of detection for each targeted LM was determined as described [83].

## STATISTICAL ANALYSIS

Data are presented as means ± standard error of the mean (SEM) using GraphPad version 9 (GraphPrism Software, San Diego, CA, USA). Determination of statistical significance was conducted on fold changes to respective time controls (days 12 and 22) using the Bonferroni’s multiple comparisons test (mixed-effects analysis). Data of mRNA expression were log_2_-transformed before statistical evaluation. Statistical significance was tested with ^*^p < 0.05, ^**^p < 0.01, and ^***^p < 0.001 for the DNCB-treated mice versus respective time control; ^+^*p* < 0.05. ^++^*p* < 0.01, and ^+++^*p* < 0.001 DEX treatment versus DNCB control at day 22; ^#^*p* < 0.05, ^##^*p* < 0.01, and ^###^*p* < 0.001 between the DNCB-treated groups at day 12 and day 22.

## FUNDING

This work was funded by the Free State of Thuringia and the European Social Fund (2019 FGR 0095) and the German Research Foundation Project Nr. 512648189 and the Open Access Publication Fund of the Thueringer Universitaets- und Landesbibliothek Jena.

### Supplementary Information

Below is the link to the electronic supplementary material.

Supplementary file1 (DOCX 118 KB)

Supplementary file2 (ZIP 118 MB)
