# Supplementary material for: Characterization of Different Inflammatory Skin Conditions in a Mouse Model of DNCB-Induced Atopic Dermatitis
Source: Inflammation. 2023 Dec 27;47(2):771–88. doi: 10.1007/s10753-023-01943-x (PMC11074229; doi:10.1007/s10753-023-01943-x)
Supplement: Supplementary file 1 — Supplementary file1 (DOCX 118 KB) [file 10753_2023_1943_MOESM1_ESM.docx]

**Supplement to the manuscript:**

Characterization of Different Inflammatory Skin Conditions in a Mouse Model of DNCB-induced Atopic Dermatitis

Rebecca Riedl ^1,2^, Annika Kühn ^2^, Yvonne Hupfer ^2^, Betty Hebecker ^2,3^, Lukas K. Peltner ^4^, Paul M. Jordan ^4,5^, Oliver Werz ^4,5^, Stefan Lorkowski ^2,3^, Cornelia Wiegand ^1^ and Maria Wallert ^2,3,*^

^1^ Jena University Hospital, Department of Dermatology, Dermatological Research Laboratory, 07747 Jena, Germany; rebecca.riedl@med.uni-jena.de (R.R.); c.wiegand@med.uni-jena.de (C.W.)

^2^ Department of Nutritional Biochemistry and Physiology, Institute of Nutritional Science, Friedrich Schiller University, 07743 Jena, Germany; rebecca.riedl@uni-jena.de (R.R.); annika.kuehn@uni-jena.de (A.K.); yvonne.hupfer@uni-jena.de (Y.H.); betty.hebecker@uni-jena.de (B.H.); stefan.lorkowski@uni-jena.de (S.L.); maria.wallert@uni-jena.de (M.W.)

^3^ Competence Cluster for Nutrition and Cardiovascular Health (nutriCARD) Halle-Jena-Leipzig, Germany

^4^ Department of Pharmaceutical/Medicinal Chemistry, Institute of Pharmacy, Friedrich Schiller University, 07743 Jena, Germany; lukas.klaus.peltner@uni-jena.de (L.K.P.); paul.jordan@uni-jena.de (P.M.J.); oliver.werz@uni-jena.de (O.W.)

^5^ Jena Center for Soft Matter (JCSM), Friedrich Schiller University, 07743 Jena, Germany

***** Correspondence: maria.wallert@uni-jena.de; Tel.: +49-3641-9-49726

**Figure S1**

**Figure S1** DNCB treatment affects lipid mediator formation in dorsal murine skin. Data are presented in pg/mg skin as means ± SEM (n=4-5 per group).

**Table S1** Commercially validated primers used with a customized 384-well RT^2^ PCR Array Plate (Qiagen) for real-time qPCR.

| Gene name | GeneGlobe ID (Qiagen) |
| --- | --- |
| *Ppih* | QT01165318 |
| *Camp* | QT00241003 |
| *Fc*$\varepsilon r$*1a* | QT00112161 |
| *Flg* | QT01195929 |
| *Lor* | QT00248192 |
| *Krt10* | QT00493241 |
| *S100a8* | QT00250264 |
| *Lcn2* | QT00113407 |
| *Defb3* | QT00103271 |
| *Ptgs2* | QT00165347 |
